# Cytochrome c oxidase deficiency caused by biallelic *SCO2* mutations in two sibs with cerebellar ataxia and progressive peripheral axonal neuropathy

**DOI:** 10.1016/j.ymgmr.2019.100528

**Published:** 2019-11-06

**Authors:** Giulia Barcia, Zahra Assouline, Alessandra Pennisi, Cyril Gitiaux, Manuel Schiff, Nathalie Boddaert, Arnold Munnich, Jean-Paul Bonnefont, Agnès Rötig

**Affiliations:** a Fédération de Génétique Médicale, Hôpital Necker Enfants Malades, Paris, France; b UMR1163, Université Paris Descartes, Sorbonne Paris Cité, Institut IMAGINE, Paris, France

We report on a homozygote missense *SCO2* mutation (p.Arg255Trp) in two sibs with cerebellar ataxia, progressive peripheral axonal neuropathy and long survival. While most patients reported so far presented with severe COX deficiency and fatal infantile cardioencephalomyopathy or Leigh syndrome, this observation extends the clinical spectrum of SCO2 mutations and prompts to consider respiratory chain deficiency as a possible cause of cerebellar ataxia with progressive peripheral axonal neuropathy.

SCO2 cytochrome *c* oxidase assembly (SCO2) is one of the cytochrome *c* oxidase (COX, Complex IV) assembly factors [1]. It is a mitochondrial metallochaperone encoded by the nuclear genome. COX catalyzes the transfer of electrons from cytochrome *c* to molecular oxygen, a reaction coupled to a proton gradient across the inner mitochondrial membrane and to ATP production. It is a multimeric complex that requires several assembly factors, including SCO2, involved in biogenesis of COX subunit II, an essential subunit for electron transfer from cytochrome *c* to the bimetallic copper center of the catalytic subunit 1. In addition, SCO2 acts as a thiol-disulfide oxidoreductase to regulate the redox state of the cysteines in SCO1.

The five mutations hitherto reported in *SCO2* are known to cause COX deficiency with fatal infantile cardioencephalomyopathy (CEMCOX1) [2,3], myopia (MYP6) [4], Leigh syndrome [5] or early-onset axonal Charcot-Marie-Tooth disease [6]. In CEMCOX1, hypertrophic cardiomyopathy is reportedly associated to developmental delay, basal ganglia and spinal cord involvement and lactic acidosis. Severe neonatal forms occasionally mimicked SMA1 phenotype [7,8]. Here we report a still different clinical presentation of *SCO2* mutations in two sibs with slowly progressive peripheral axonal neuropathy, cerebellar ataxia and no cardiomyopathy.

Patient 1, the first child of distantly related parents of West African origin, was born after a term pregnancy and normal delivery (birthweight: 3010 g, height: 48 cm, OFC: 33,5 cm). He did well in his first 18 mths of life, smiled aged 4 mths and could sit unaided aged 9 mths. He walked aged 19 mths but his gait was unstable, with frequent falls. At 2 yrs., he could walk a few steps with gait ataxia and tremor. Clinical examination revealed a severe distal myoatrophy of the four limbs, with lack of ankle reflexes, normal patellar reflexes, cerebellar syndrome, dysmetry and tremor. No pyramidal or extra-pyramidal syndrome was noted. He had convergent strabismus, ophthalmoplegia but normal eye fundus and no lid ptosis. At age 6 yrs., brain MRI showed bilateral cerebellar vermis atrophy with T2 hyper-intensities of the posterior white matter and no evidence of basal ganglia involvement. At age 8 yrs., he behaved relatively well but he had no speech and attended a special school. Heart ultrasounds were consistently normal (left posterior wall thickness < 6 mm, shortening fraction: 35%). Electro-diagnostic studies showed severe axonal neuropathy and auditory evoked potentials detected an altered central conduction ( + 5 SD, left and right auditory threshold: 30 dB). EEG and ERG were unremarkable.

Metabolic work up showed elevated levels of plasma lactate (2.7–3.1 mmol/L, normal values below 2 mmol/L), CSF lactate (3.2 mmol/L, normal values below 2.4 mmol/L) and L/P ratios (23, normal below 10). Plasma and urinary amino acids and organic acids were normal. Spectrophotometric analysis of respiratory chain enzymes on skeletal muscle homogenate showed isolated COX deficiency (141 nmol/mn/mg proteins; controls: 609 ± 202 nmol/mn/mg proteins; COX/citrate synthase: 0.5, control values: 1.5 ± 0.5). COX activity was also deficient in muscle mitochondria-enriched fractions (COX: 1233 nmol/mn/mg proteins, control values: 3151 ± 760 nmol/mn/mg, COX/citrate synthase: 0.5, control values = 1.3 ± 0.3). Consistently, spectrophotometric analyses and activity ratios of mitochondrial enzyme activities in cultured skin fibroblasts detected an isolated COX deficiency (5 nmol /min/mg proteins, control values: 47–182 nmol/mn/mg prot; COX/Citrate synthase: 0.6, control values: 1.14–1.54).

Patient 2, his younger sister, could walk unaided aged 16 mths and developed a cerebellar syndrome, with ataxic gait, mild tremor and distal muscular atrophy aged 28 mths. She had strabismus, abnormal eye movements and weak, yet present deep tendon reflexes. She had an opened, playful personality; she could say a few words and attended normal school. Her presentation was less severe and her clinical course more slowly progressive compared to her brother. Heart ultrasound at 6 yrs. was normal and brain MRI showed cerebellar atrophy with posterior hyperintensities of the white matter. COX activity was defective in her circulating lymphocytes (COX = 54 nmol/min/mg proteins, control values: 96–162 nmol/mn/mg proteins) and cultured skin fibroblasts (COX: 61 nmol/min/mg proteins, control values: 47–182 nmol/mn/mg proteins; COX/CS = 0.78, control values: 1.14–1.54).

Targeted exome sequencing detected a homozygote c.763C > T variation in the *SCO2* gene (NM_001169109.1) of both patients (p.Arg255Trp). Their parents were heterozygotes for this variant and no other variation was detected on sequencing the entire mitochondrial genome. This variation is predicted to be damaging by the Alamut Visual software (https://www.interactive-biosoftware.com/alamut-visual/), deleterious by the SIFT software (score: 0, median: 3.29), disease causing by MutationTaster: (*p*-value: 0.999), and probably damaging by Polyphen-2, with a score of 0.952 (sensitivity: 0.64; specificity: 0.92). This variation has been reported as a SNP (rs112793292) with a low frequency (ExAC: *T* = 0.00080% and MAF/MinorAlleleCount *T* < 0.01/9) and is considered of “uncertain significance” in CLINVar. The Arg255 is conserved between human and baker yeast and lies in a conserved region of the protein. Arg255 is located in the α4 helix of the SCO2 protein [9]. Based on the 3D structure of human SCO2 protein (pdb code: 2rli), this residue is involved in a H bond with the Ala259 residue (distance 1.91 Å) and the Arg255Trp substitution is expected to create an additional H bond with Ser251 (H bond distance 3.01) and to increase the H bond distance with Ala259 (Fig. 1).

**Fig. 1:**
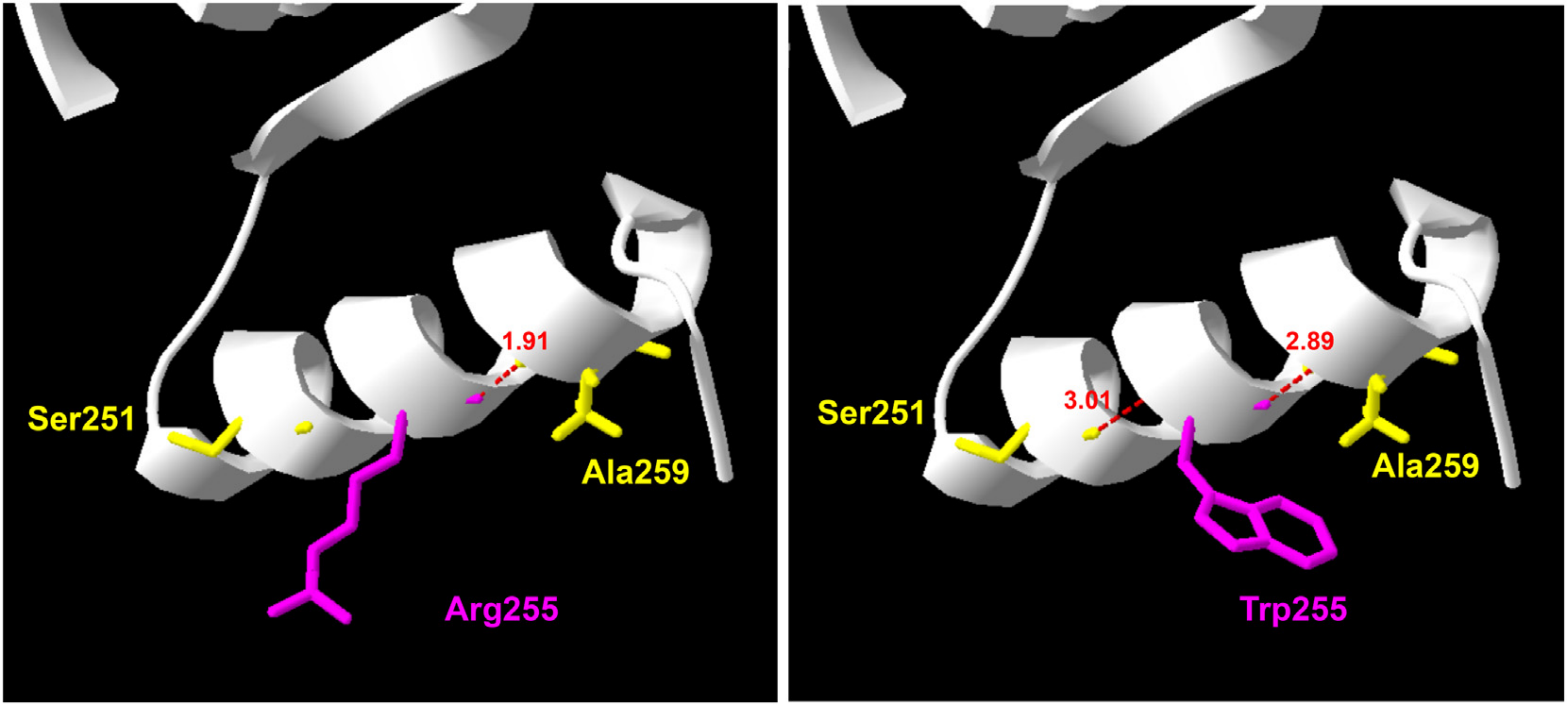
Localization of the Arg255 Trp255 substitution (in pink) in a crystallized model of human SCO2 protein. The H bonds are shown in red. The Arg255Trp substitution is expected to create an additional H bond with Ser251 (yellow) (H bond distance 3.01 Å) and to increase the H bond distance with Ala259 (yellow). (For interpretation of the references to colour in this figure legend, the reader is referred to the web version of this article.)

The five pathogenic variants reported so far in the human SCO2 protein have been located on the 3D structure of the protein [9]. Interestingly, another Arg to Trp substitution, located upstream of the one reported here (Arg171Trp) [5], was expected to produce the loss of salt bridges between Arg171 and two conserved charged residues in loop 5 (Asp168 and Glu170). Similarly, the closest reported mutation (Ser225Phe) [2], was expected to alter the copper binding properties of SCO2. Taken together and based on previous structural studies, we believe that the Arg255Trp variation reported here is the disease causing mutation in our two COX deficient sibs and that this pathogenic variation likely destabilizes the protein fold, rendering the protein more susceptible to aggregation and/or degradation.

Owing to the severity of cardiomyopathy and/or Leigh disease, most cases of *SCO2* mutations reported so far exhibited an early onset and a rapidly fatal course [2,5]. Interestingly, the two sibs reported here presented with cerebellar ataxia and slowly progressive peripheral axonal neuropathy, neither retinal nor heart involvement and a long survival. This observation, reminiscent of the two cases reported by Rebello et al. [6], supports the remarkable, yet unexplained clinical variability of SCO2 mutations and prompts to consider respiratory chain deficiency as a possible cause of cerebellar ataxia with progressive peripheral axonal neuropathy.

## References

[1] Leary S.C., Sasarman F., Nishimura T., Shoubridge E.A. (2009). Human SCO2 is required for the synthesis of CO II and as a thiol-disulphide oxidoreductase for SCO1. Hum. Mol. Genet..

[2] Papadopoulou L.C., Sue C.M., Davidson M.M., Tanji K., Nishino I., Sadlock J.E., Krishna S., Walker W., Selby J., Glerum D.M., Coster R.V., Lyon G., Scalais E., Lebel R., Kaplan P., Shanske S., De Vivo D.C., Bonilla E., Hirano M., DiMauro S., Schon E.A. (1999). Fatal infantile cardioencephalomyopathy with COX deficiency and mutations in SCO2, a COX assembly gene. Nat. Genet..

[3] Sacconi S., Salviati L., Sue C.M., Shanske S., Davidson M.M., Bonilla E., Naini A.B., De Vivo D.C., DiMauro S. (2003). Mutation screening in patients with isolated cytochrome c oxidase deficiency. Pediatr. Res..

[4] Tran-Viet K.N., Powell C., Barathi V.A., Klemm T., Maurer-Stroh S., Limviphuvadh V., Soler V., Ho C., Yanovitch T., Schneider G., Li Y.J., Nading E., Metlapally R., Saw S.M., Goh L., Rozen S., Young T.L. (2013). Mutations in SCO2 are associated with autosomal-dominant high-grade myopia. Am. J. Hum. Genet..

[5] Jaksch M., Paret C., Stucka R., Horn N., Muller-Hocker J., Horvath R., Trepesch N., Stecker G., Freisinger P., Thirion C., Muller J., Lunkwitz R., Rodel G., Shoubridge E.A., Lochmuller H. (2001). Cytochrome c oxidase deficiency due to mutations in SCO2, encoding a mitochondrial copper-binding protein, is rescued by copper in human myoblasts. Hum. Mol. Genet..

[6] Rebelo A.P., Saade D., Pereira C.V., Farooq A., Huff T.C., Abreu L., Moraes C.T., Mnatsakanova D., Mathews K., Yang H., Schon E.A., Zuchner S., Shy M.E. (2019). SCO2 mutations cause early-onset axonal Charcot-Marie-tooth disease associated with cellular copper deficiency. Brain.

[7] Tarnopolsky M.A., Bourgeois J.M., Fu M.H., Kataeva G., Shah J., Simon D.K., Mahoney D., Johns D., MacKay N., Robinson B.H. (2004). Novel SCO2 mutation (G1521A) presenting as a spinal muscular atrophy type I phenotype. Am. J. Med. Genet. A.

[8] Salviati L., Sacconi S., Rasalan M.M., Kronn D.F., Braun A., Canoll P., Davidson M., Shanske S., Bonilla E., Hays A.P., Schon E.A., DiMauro S. (2002). Cytochrome c oxidase deficiency due to a novel SCO2 mutation mimics Werdnig-Hoffmann disease. Arch. Neurol..

[9] Banci L., Bertini I., Ciofi-Baffoni S., Gerothanassis I.P., Leontari I., Martinelli M., Wang S. (2007). A structural characterization of human SCO2. Structure.

